# The impact of radiofrequency ablation on overall survival in patients with hilar cholangiocarcinoma: an analysis using prediction interval

**DOI:** 10.1097/JS9.0000000000003529

**Published:** 2025-09-23

**Authors:** Liang Chen, Xianghui Fu, Jiacheng Chen

**Affiliations:** a Department of Hepatobiliary & Pancreatic Surgery, Hainan General Hospital, Hainan Affiliated Hospital of Hainan Medical University, Haikou, Hainan Province, China; b Department of Gastrointestinal Surgery, Hainan General Hospital, Hainan Affiliated Hospital of Hainan Medical University, Haikou, Hainan Province, China

*Dear Editor,*

We have read with great interest the study conducted by Zhou *et al*^[1]^, which investigated the safety and efficacy of radiofrequency ablation (RFA) on patients with hilar cholangiocarcinoma. Through a thorough analysis of 11 studies encompassing a total of 874 patients, the authors revealed that, in comparison to those in the stent-only group, patients in the RFA combined with stent group exhibited significantly superior overall survival and stent patency, while comparable adverse events were noted. The authors deserve commendation for their timely conducted synthetic analysis, which addresses the clinical significance of RFA in this challenging malignancy.

However, as the primary endpoint of this meta-analysis, we found that the overall survival benefit conferred by the combination of RFA and stent placement was only evident in the observational study subgroup, but not in the randomized controlled study subgroup. Although the heterogeneity (*I*^2^ = 34%) of the overall meta-analysis result was acceptable, this discrepancy still raised our concerns about the stability and reliability of this crucial result. To determine whether the existing evidence clearly supports that RFA combined with stent placement can improve the overall survival of these patients compared to stent placement alone, we calculated a 95% prediction interval (PI) based on the raw data from the meta-analysis. This PI was intended to cover the expected range of effects around the summary estimate. By taking into account both the variability between studies and the uncertainty associated with the summary effect, a PI indicates the range within which we would expect approximately 95% of the true effect sizes of similar future studies to fall^[2,3]^. The PI was computed using R software (version 4.3.1; R Core Team, URL: http://www.R-project.org/). The calculated PI range for overall survival was from 0.44 to 1.24 (Fig. 1). Since this interval includes the null value of 1, it implies a high degree of uncertainty regarding the pooled effect and emphasizes the need for cautious interpretation of this finding.

**Figure 1.**
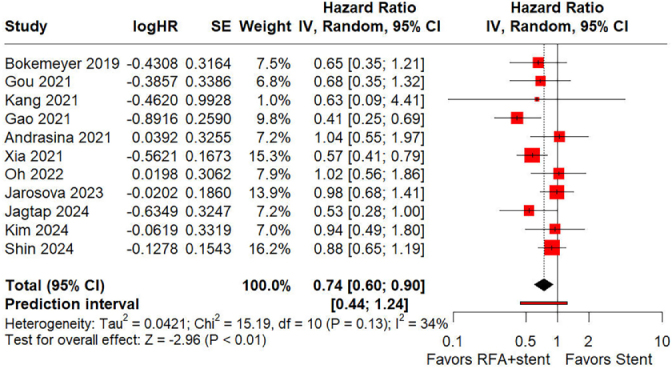
Forest plot displaying pooled hazard ratio (HR) with 95% confidence interval (CI) and 95% prediction interval (PI) for the association between radiofrequency ablation (RFA) and overall survival in patients with hilar cholangiocarcinoma.

In addition, relying solely on visual assessment of the funnel plots to test for publication bias in the pooled outcomes was not convincing. According to Cochrane’s guidelines, Begg’s, and Egger’s tests are recommended to address this concern^[4,5]^.

Overall, we wish to convey our gratitude to Zhou and colleagues for their efforts in examining the role of RFA in hilar cholangiocarcinoma. While additional confirmatory evidence is needed to validate some of their conclusions, this research highlights the potential importance of this approach in enhancing the short-term and long-term outcomes of these patients.

This study is compliant with the TITAN Guidelines 2025^[6]^.

## Data Availability

No original data used in this letter to the editor.

## Research registration unique identifying number (UIN)

No need for this letter to the editor.
